# Systematic Review of the Proposed Associations between Physical Exercise and Creative Thinking

**DOI:** 10.5964/ejop.v15i4.1773

**Published:** 2019-12-19

**Authors:** Emily Frith, Seungho Ryu, Minsoo Kang, Paul D. Loprinzi

**Affiliations:** a Exercise & Memory Laboratory, Department of Health, Exercise Science and Recreation Management, The University of Mississippi, University, MS, USA; b Health and Sports Analytics Laboratory, Department of Health, Exercise Science and Recreation Management, The University of Mississippi, University, MS, USA; University of Liverpool, UK

**Keywords:** cognition, exercise psychology, health promotion, innovation, mental health, physical activity, research methods

## Abstract

The objective of this study was to evaluate the association between physical exercise and creative thinking. A systematic review approach was employed by searching PubMed, Google Scholar and PsychInfo databases. Among the evaluated 13 studies, 92% indicated a beneficial relationship. However, 77% were vulnerable to moderate-high risk for methodological bias, suggesting adherence to standardized and controlled research initiatives should be promoted. There appears to be weak to modest support for acute, moderate-intensity exercise to benefit creativity. Exercise timing relative to creativity assessment protocols should be addressed and further detailed. Creativity scoring procedures must be refined, and an increased focus on the motivational components of exercise may help guide researchers in measuring creative thoughts and behavior. Broader concluding claims that creativity, in general, is improved or impaired by exercise, is as problematic as sweeping statements that exercise improves or impairs a measure as dynamic as intelligence. Scientific inquiries must specify precisely which outcome characteristics are changing in line with research interventions. This review identifies several fallible linkages between physical activity and creativity. Too few studies were conducted on strong methodological foundations, perpetuating the risk for undermining or inaccurately inflating the potential association between exercise and creative thinking behavior.

Scientific inquiry in any field is difficult when the parameter beneath the lens of empirical scrutiny is difficult to both operationalize and localize. Researchers have attempted to define creativity as a broad construct that encompasses “the degree of novelty of which the person is capable, or which he [or she] habitually exhibits” (Guilford, 1950). More recently, the intricate processes of creativity have been posited to correspond with “our ability to change existing patterns, break with the present, and build something new” (Dietrich & Kanso, 2010). The variegated outcomes of such creative processes have also been described as products which “can be tested in terms of the frequency of uncommon, yet acceptable, responses to items” (Guilford, 1950). Further, a theme of societal relevance, or value, is proposed as a crucial standard for creative production, as “the creative work is a novel work that is accepted as tenable or useful or satisfying by a group in some point in time” (Stein, 1953).

Despite the volume and remarkable adaptability of creative exposition, Guilford revolutionized empirical creativity assessment with a push to evaluate creative divergent thinking (Guilford, 1950). The examination of divergent thinking, or the creative act of generating multiple solutions from a single stimulus (Berkowitz, 2014), continued to serve as a pervasive staple for the best measurement practices in creativity studies for decades, and is still widely investigated in modern research. Indeed, it is well established that divergent thinking is a tool for predicting creative potential (Runco, 2008). However, it is certainly not the only tool that should be wielded by scientists searching for causal relationships. For example, convergent thinking, or solving a task with one correct solution, is also suggested to play a large role in explaining the nature of creative thought (Berkowitz, 2014). By the late 1990’s, creativity research diverted from a narrowed focus on the evaluation of divergent thinking, and began to encompass a broader range of scientific analysis, including neuroscientific correlates, personality, insight, and other systems-based approaches exposing important creativity measurement outcomes (Csikszentmihalyi, 1999; Mumford, 2003).

Unfortunately, as creativity research efforts in psychological and neurobiological disciplines appear to be making headway towards the practical conceptualization of such an untenable construct, creativity research in exercise science and health promotion is stunted. The lack of experimental work on this topic is staggering, with only 13 research studies investigating the associations between physical exercise and quantifiable creative products (Blanchette, Ramocki, O'del, & Casey, 2005; Colzato et al., 2013; Curnow & Turner, 1992; Gondola, 1986, 1987; Gondola & Tuckman, 1985; Herman-Tofler & Tuckman, 1998; Hinkle, Tuckman, & Sampson, 1993; Oppezzo & Schwartz, 2014; Ramocki, 2002; Steinberg et al., 1997; Tuckman & Hinkle, 1986; Zhou, Zhang, Hommel, & Zhang, 2017). Over half of this body of literature was published prior to the millennium, and, as demonstrated herein, the vast majority lack sound rationale and methodological quality.

Following the systematic review framework detailed elsewhere (Murad et al., 2014), this systematic review will provide a detailed synthesis of the exercise and creativity work accomplished thus far. The dearth of unbiased research on exercise and creativity is a critical issue, which must be prevented for future development in this area to continue unencumbered by obstruction, or even absence, of meaningful evidence to answer the pervasive question, “Does exercise influence creative potential?” Therefore, a secondary aim of this review is to direct future experimentation towards more informed, and applicable, methods of inquiry, and provide direction for identifying prudent questions worth answering in the field.

## Method

### Inclusion Criteria

Research studies were included if they utilized an experimental study design, were published in English, indexed in PubMed, Google Scholar and PsycInfo, and specifically evaluated the influence of acute or habitual physical exercise on creativity in children or adults, of either gender and with no known psychological or physical limitations or preexisting pathology that would prevent them from being classified as healthy at baseline. Any exercise intervention (acute or chronic laboratory or free-living physical activities) coupled with either an active or traditional control group (no exercise) was considered.

#### Outcome measure

Cognitive creativity (analogy, convergent thinking, divergent thinking, insight, metaphors, and problem-solving).

### Exclusion Criteria

Research studies were excluded if no exercise intervention was employed, self-report questionnaires of creative strengths and abilities were not accompanied by an observable laboratory measure of creative potential (McCutcheon, 1982), or if creativity was not the outcome variable (Hallihan & Shu, 2011). Additionally, articles were excluded if the study population was comprised of nonhuman subjects.

### Search Strategy

The following databases were searched between 1 January 2018 and 10 January 2018: PubMed, PsychInfo, and Google Scholar. MeSH keyword terms included exercise, physical exercise, physical activity, creativity, exercise and creativity, physical exercise and creativity, and physical activity and creativity. Subordinate terms included convergent thinking, divergent thinking, and insight problem-solving.

**Figure 1:**
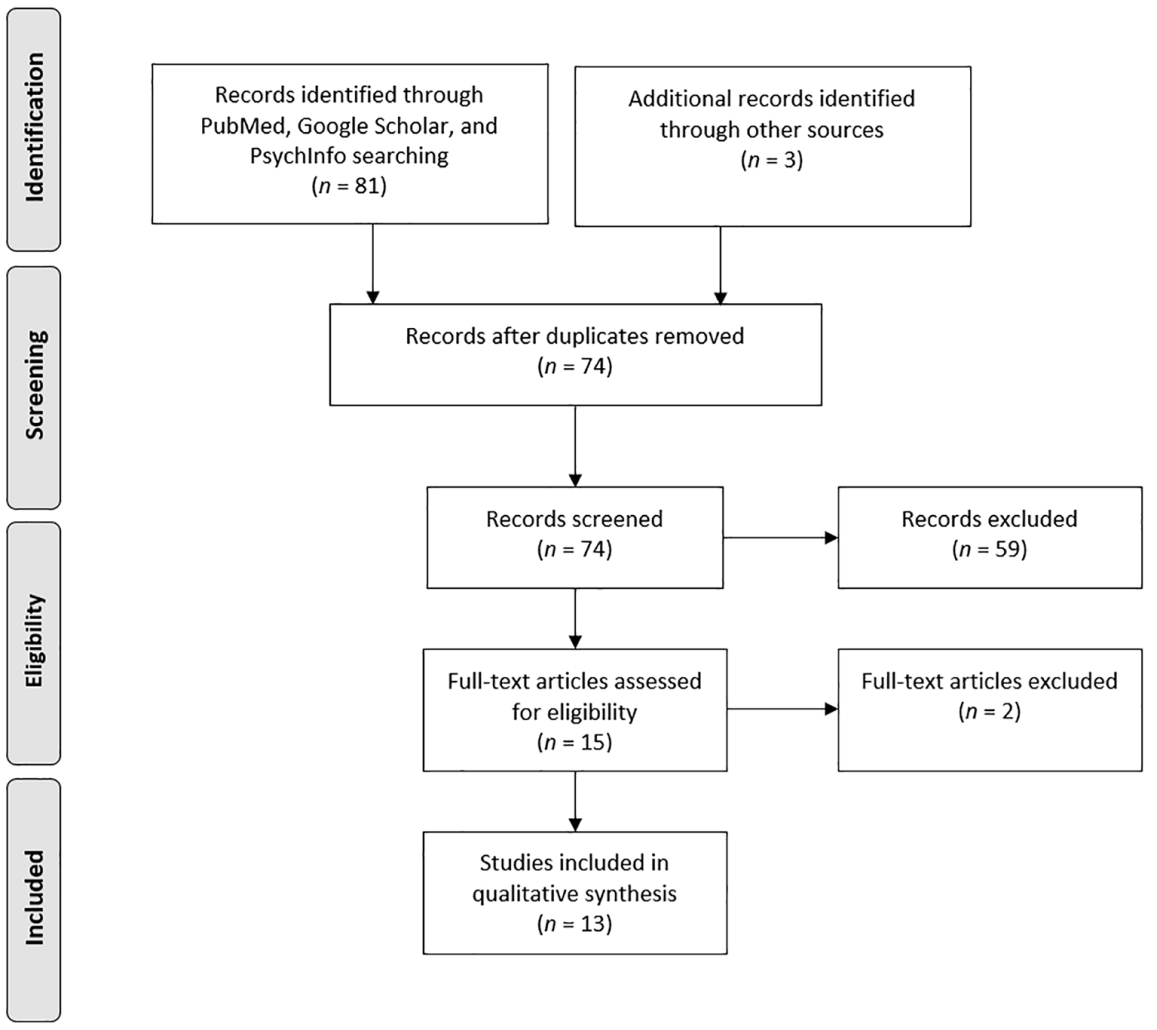
Flow diagram of the extracted studies.

See Figure 1 for a flow diagram of the extracted studies from the computerized search. In total, 13 articles met the study criteria (Figure 1).

### Quality Evaluation

Risk of bias/study quality was evaluated for each article using a checklist developed specifically for this study. The following checklist includes seven items with a yes (1) or no (0) response option and was constructed in accordance with the Cochrane Risk of Bias Tool (Higgins & Green, 2008). This risk of bias checklist, as well as this entire systematic review, also adhered to the PRISMA checklist for reporting systematic reviews (except for Item 5, prospective registration of the systematic review). Two of the authors independently scored each study based on this checklist. Discrepancies were discussed until agreement was made. In situations when consensus did not occur, the two remaining authors were consulted, which facilitated consensus for all risk of bias items. The risk of bias items are as follows:

1. Was the physical activity manipulation controlled (e.g., completed in a laboratory setting, standardized by duration and intensity, and for interaction with other participants if administered in a group context)?
2. Was there evidence of reliability for the creativity measure(s) utilized?
3. Was there evidence of validity for the creativity measure(s) utilized?
4. Were creativity scoring and evaluation procedures robust to bias (e.g., blinded scoring completed by multiple researchers, provision of strong interrater reliability, and detailed or referenced?
5. Were random group assignment and/or counterbalancing procedures appropriate (e.g., were participants assigned to groups based on course enrollment, rather than random selection and were the order of creativity assessments randomized to ensure resistance to temporal artifacts or learning effects?) for the study design?
6. Did the intervention use a non-exercise control group or condition?
7. Were statistically appropriate/acceptable methods of data analysis used?
8. Were point estimates, standard deviations, confidence intervals, and/or effect sizes reported?

Items 2 and 3 required each manuscript to include an explicit description of evidence for reliability and validity of the creativity outcome assessments employed. The manuscript earned a ‘no’ (0) score for missing details regarding reliable/valid measures, which may have been utilized in some experiments, but were not adequately detailed per our quality evaluation criteria. A ‘no’ score for Item 7 was awarded to manuscripts that failed to use reasonable statistical methods for post-hoc analysis of outcomes. Statistically inappropriate decisions included reporting Pearson correlation coefficients for interrater reliability, failure to use the appropriate statistical tests and computing unpaired analyses of treatment effects on individual differences as a result of chronic training studies. Item 8 identified articles that neglected to report point estimates, confidence intervals, standard deviations, and/or effect sizes. The authors may have computed these values, but if all statistical results were not included in the publication, a ‘no’ score was given for Item 8.

The 13 included research studies were classified into categories based on cut points reflecting the degree of methodological bias considered for each individual study. Studies with a score of 6–8 (three studies) were classified as having low risk of bias. Studies with a score of 3–5 (eight studies) were classified as having moderate risk of bias. Studies with a score of 0–2 (two studies) were classified as having a high risk of bias (Table 1).

**Table 1:** Risk of Bias Assessment

Study	Risk of Bias Item								Total
	1	2	3	4	5	6	7	8	
Blanchette et al. (2005)		x	x	x	x	x			5
Colzato et al. (2013)	x	x	x	x	x	x		x	7
Curnow and Turner (1992)	x	x	x	x			x		5
Gondola and Tuckman (1985) [pilot study]		x	x	x					3
Gondola (1986)		x	x						2
Gondola (1987)		x	x				x		3
Herman-Tofler and Tuckman (1998)		x	x	x				x	4
Hinkle et al. (1993)		x	x	x	x		x		5
Oppezzo and Schwartz (2014)	x	x	x	x	x	x	x	x	8
Ramocki (2002)						x			1
Steinberg et al. (1997)		x	x			x	x		4
Tuckman and Hinkle (1986)		x	x				x		3
Zhou et al. (2017)	x			x	x	x	x	x	6

*Note*. **Item 1:** Was the physical activity manipulation controlled (e.g., completed in a laboratory setting, standardized by duration and intensity, and for interaction with other participants if administered in a group context)? **Item 2:** Was there evidence of reliability for the creativity measure(s) utilized? **Item 3:** Was there evidence of validity for the creativity measure(s) utilized? **Item 4:** Were creativity scoring and evaluation procedures robust to bias (e.g., blinded scoring completed by multiple researchers, provision of strong interrater reliability, and detailed or referenced? **Item 5:** Were random group assignment and/or counterbalancing procedures appropriate (e.g., were participants assigned to groups based on course enrollment, rather than random selection and were the order of creativity assessments randomized to ensure resistance to temporal artifacts or learning effects?) for the study design? **Item 6:** Did the intervention use a non-exercise control group or condition? **Item 7:** Were statistically appropriate/acceptable methods of data analysis used? **Item 8:** Were point estimates, standard deviations, confidence intervals, and/or effect sizes reported?

### Data Extraction

A data extraction table for the included research studies was created to provide a brief description of author names and publication date, sample characteristics, research design, creativity measures used and length of creativity assessment period, relevant creativity parameters assessed, exercise modality, intensity and duration, methods used for scoring creative products, as well as study outcomes and conclusions (Table 2).

**Table 2:** Extraction Table of the Evaluated Studies

Study / Sample Characteristics	Research Design	Creativity Measures (duration)	Creativity Parameters Assessed	Exercise Modality (intensity and duration)	Scoring	Outcomes and Conclusions
Blanchette et al. (2005)						
*N* = 60, 30 males; 30 females; Age: 18–27 (*M* = 20)	within-subject	1. TTCT Figural Tests A and B (10 minutes each form) 2. Creative Strengths Questionnaire	Abstractness of titles, fluency, originality, elaboration, resistance to premature closure per TTCT scoring guide	Acute exercise protocol; primarily aerobic; self-selected (moderate: 30-minute)	Four independent authors scored all of these anonymous instruments in random order. Interrater reliability was high with Pearson Correlation medians of .818 (range .766–.886) for H1, .850 (range .789–.870) for H2, and .826 (range .781–.917) for H3.	Creative potential was elevated immediately post-exercise, relative to control (*p* < .001) Creative potential was elevated 2-hrs post-exercise, relative to control (*p* < .001) No statistically significant temporal differences were determined Between the two exercise conditions (*p* = .251)
Colzato et al. (2013)						
*N* = 96 (Age: *M* = 21) Experimental Group: 48 habitual exercisers Control Group: 48 inactive individuals	between-subjects cross-over design	30 RAT triads RAT (10 triads per condition) 3 AUT items (1 item per condition)	Flexibility, fluency, originality, elaboration	Cycle ergometer ((rest (6-minute), (moderate (6-minute), (and intense (6-minute)) exercise (12-minute total cycling time) Creativity was assessed during exercise for half of the participants in each group (24-minutes total protocol), and immediately after for the remaining half (36-minute total protocol)	RAT scored numerically via an index of total correct responses AUT scoring was completed by two independent raters for the divergent thinking measure-no indication if participant responses were blinded to raters Cronbach’s alpha scores for fluency, flexibility, originality and elaboration ranged from 0.74 to 1.00. We assume the authors intended to report inter-rater reliability	Intense exercise was associated with reductions in convergent thinking among inactive participants, compared to engaging in moderate exercise (*p* = .002) and rest (*p* = .029). Creative flexibility on the AUT was higher at rest, than for intense exercise (*p* = .011) for both groups. There was no difference in AUT flexibility performance during rest or moderate-intensity exercise for both groups (*p* = .150).
Curnow and Turner (1992)						
*N* = 46, 35 females; Age: 18–24 (*M* = 19) A) Music and Exercise B) Exercise Only C) Music Only D) Control Group (no exercise-no music) (no sample size reported for each separate group)	between-subjects	TTCT Figural tests A (pre) and B (3-minute post condition)	Fluency, originality and elaboration	Cycle ergometer (20-minute submaximal workload of 150 kpm at a rate of 55 rpm	The Scholastic Testing Service, Earth City, MO scored the assessments. However, no inter-rater reliability was reported.	There were no statistically significant differences between groups for any creativity measure assessed.
Gondola and Tuckman (1985)						
Control (no PA): *n* = 23 Experimental Group: *n* = 26	Mixed model	AUT, Match Sticks and Consequences	Pre-study and post-study chronic creativity (before exercising) Match Sticks, Obvious Consequences, Remote Consequences and AUT	8-week chronic training study (20-minute run for 16 sessions-2× per week)	Followed scoring guides for convergent and divergent thinking measures Did not detail how the scoring was completed, or if scoring was conducted by internal or external raters. No inter-rater reliability was reported	The experimental group outperformed the control group on the AUT (*p* < .01) No additional differences were determined for the included creativity assessments
Gondola (1986)						
Experimental Group 1: *n* = 23 Experimental Group 2: *n* = 19 Co-ed undergraduates (no other demographics reported) Control: no sample size reported	Mixed model	AUT, Match Sticks and Consequences	Group 1 and 2: Pre-study and post-study chronic creativity (before exercising) Match Sticks, Obvious Consequences, Remote Consequences Group 2: Acute Creativity (Match Sticks, Obvious Consequences, Remote Consequences and AUT) measured pre-and post-exercise for session 1	Group 1: 8-week chronic training study: 20-minute run for 16 sessions (2× per week) Group 2: 6-week chronic training study: 20-minute run for 12 sessions (2× per week)	Scoring was completed by the author and one assistant. No inter-rater reliability was reported	Both experimental groups performed better on the AUT relative to controls (*p* < .001). Group 2 scored higher on Remote Consequences than the other two groups (*p* < .01). Pre and post-acute creativity scores for Remote Consequences and the AUT were statistically significantly different for Group 2 (*p* < .001).
Gondola (1987)						
*N* = 37 females; Age: 19–35 (*M* = 23) Experimental Group: *n* = 21 Control Group (no PA): *n* = 16	Mixed-model	AUT and Consequences	Acute creativity assessed at baseline and 5-minute post-exercise 1 week later (two visits)	20-minute moderate-to-vigorous aerobic dance	No description of scoring methods was provided for replication. No inter-rater reliability was reported	The experimental group scored higher on the AUT than the control group (*p* < .0001) The experimental group scored higher on the Remote Consequences than the control group (*p* < .01)
Herman-Tofler and Tuckman (1998)						
*N* = 52 third graders randomized into an Experimental (aerobic exercise physical education) or Control Group (traditional physical education) No sample size per group was reported.	Mixed-Model	TTCT Figural Test-Forms A (vertical parallel lines) and B (circles) Time to complete the creativity assessments was not reported	Picture construction-original and detailed stories; multiple associations and divergent thinking	3 aerobic exercise sessions per week for 8 weeks	Scoring per the TTCT manual TTCT test-retest reliability coefficients were reported for the figural test forms (0.71–0.85). No inter-rater reliability was reported	The aerobic exercise group achieved increased figural fluency scores pre-to-post-intervention, compared to the control group (*p* = .04) Aerobic power (measured via an 800-m run) was not statistically significantly different from pre-intervention to post-intervention (*p* = .266)
Hinkle et al. (1993)						
*N* = 85 Experimental Group: *n* = 42; 20 males; 22 females Control Group: *n* = 43; 24 males; 19 females (Age: *M* = 13)	Mixed-Model	Figural and Verbal versions of the TTCT tested in a group setting	Verbal: divergent thinking, fantasy, unique thinking Figural: elaboration, fluency, originality, and breaking set	Five outdoor running sessions per week for 8 weeks (no duration provided)	No description of scoring methods was provided for replication All creativity assessments were scored by one independent rater. Therefore, no inter-rater reliability could be reported	Pre-to-post scores for fluency, flexibility, and originality were marginally higher in the treatment group compared to controls (*p* < .05) Females, irrespective of condition assignment, achieved marginally higher increases in verbal flexibility, verbal originality, and figural elaboration (*p* < .05).
Oppezzo and Schwartz (2014)						
Experiment 1: *N* = 48 undergraduate psychology students Experiment 2: *N* = 48; sit-sit; sit-tread; tread-sit conditions Experiment 3: *N* = 40; sit-sit; sit-walk; walk-sit; and walk-walk Experiment 4: *N* = 40; sit inside; walk inside; sit outside; walk outside	1) within-subject 2) between-subjects 3) between-subjects 4) between-subjects (2 × 2 design)	1) AUT (4-minute × 2 tasks consisting of 6 items total) and RAT (4-minute for 16 triads) 2) AUT (4-minute × 2 tasks consisting of 6 items total) × 2 3) AUT (same as above) 4) BSE (5-minute × 3 tasks-16-minute total session)	Ideation, novelty, appropriate uses, appropriate novelty, and non-repetitive uses 3 only) alfresco code (“outdoor” ideas) 4 only) analogy production coded for appropriateness, novelty, and high-quality responses, further determined by degree of detail and semantic distance	1) 12-minute seated followed by 12-minute treadmill walking 2) 8-minute of condition; 8-minute of complementary condition (i.e., 8-minute sit followed by 8-minute tread) 3) 16-minute seated indoors; 8-minutes seated indoors and 8-minute walking outdoors or 8-minute walking outdoors and 8-minute seated indoors; 16-minute walking outdoors	All divergent thinking parameters were subject to a-priori defined, researcher operationalizations of creativity Analogies were further scored using Amabile’s (1996) consensual assessment technique. Interrater reliability for the AUT was reported as r = .73. for Experiments #1 and #2, *r* = .74 for Experiment #3, and *r* = 1.0 for detail level and *r* = .98 for semantic distance in Experiment #4.	1) RAT performance decreased when walking (*p* = .03), while AUT performance increased when walking (*p* < .001). 2) The order of walking (before or after sitting) did not yield statistically significant differences (*p* = .975) at the end of the bout. Decreased ideation on the AUT was determined from time-point 1 to time-point 2 in the tread-sit condition (*p* = .016). Walking was associated with higher creativity performance on the AUT than sitting (*p* < .001). 3) Walking once was not statistically different than walking at both time-points on the AUT (*p* = .253) Walking at both time-points resulted in a similar level of maintained creativity performance on the AUT across time (*p* = .507) Sitting after walking mirrored the findings of experiment 2. Sitting after walking was associated with comparable creativity performance on the AUT as that achieved during walking (*p* = .335). 4) Walking was associated with higher-quality, novel analogies relative to individuals who sat. Being outdoors was independently related to novelty, albeit perhaps of lower-quality responses
Ramocki (2002)						
*N* = 31 Experimental Group: *n* = 15 Control Group (no PA): *n* = 16 Age 20–40	between-subjects	Baseline: AUT (20-minute), game development, (40-minute) Post: metaphors (20-minute), planning a party (40-minute)	Creative fluency, flexibility, novelty (categorical), and global creativity (rank-ordered)	One-hour of self-selected vigorous-intensity physical activity for experimental group	Double-blinded scoring completed by three faculty and three student-raters (also participants in the study). Kendall's Coefficients for the pretest assessment were *W* = .66 for the subject judges, *W* = .62 for the faculty judges, and *W* = .56 for all six judges combined. Kendall's Coefficient for the posttest assessment was *W* = .59 for the subject judges, *W* = .73 for the faculty judges, and *W* = .61 for all six judges combined.	Only the mean change in pre- to post-fluency was statistically significant for the experimental group (*p* < .01).
Steinberg et al. (1997)						
*N* = 63 Aerobic Exercise Group: 15 males; 16 females; age range 19–54; median age range 25–29 Aerobic Dance Group: 4 males; 28 females; age range 19–59; median age range 20–24 Four students were lost to attrition.	Mixed Model	Unusual Uses Test of Creative Thinking (Tin Cans and Cardboard Boxes-5-minute per item)	Fluency, flexibility, and originality	17 minutes of aerobic exercise defined as high-impact 21.7 minutes of aerobic dance defined as low-impact A control condition was completed (counterbalanced order), consisting of a neutral video matched to exercise duration	Scoring of unusual uses was based on ratings summed across a four-point scale Inter-rater reliability was reported between two independent raters at *r* = .89.	Flexibility was marginally higher in the exercise condition, compared to the video condition (*p* < .05) Although favorable improvements in mood occurred with exercise (*p* < .001), mood failed to contribute to effects on creativity (> .05)
Tuckman and Hinkle (1986)						
*N* = 154 *n* = 48 4^th^ graders (Age: *M* = 9) *n* = 53 5^th^ graders (Age: *M* = 10) *n* = 53 6^th^ graders (Age: *M* = 11) Number of participants in Experimental and control groups was not specified	Mixed Model	AUT (10 items-no duration provided)	No mention of specific creativity parameters was provided	Three outdoor running sessions per week (30-minute each session) for 12 weeks Active control group participated in regular physical education class activities	No procedures for scoring methods were reported. Thus, no inter-rater reliability was reported	The experimental group outperformed the control group on the AUT (*p* < .001) Boys in the experimental group achieved marginally higher AUT scores than girls following posttest analyses (*p* < .05)
Zhou et al. (2017) (Study 2a and 2b were excluded, as these did not evaluate exercise)						
Study 1a. *N* = 63, 21 males and 42 females, Age: *M* = 21.25 [Study 1b. Same participants]	within-subject	1a) DIT divergent thinking task 1b) CIT divergent thinking task (10 trails; 1-minute allocated to each trial)	1a) Scored task completion and task novelty 1b) Scored fluency, flexibility, and novelty	Study 1 and 1b) standing, constrained walking-Figure-of-8 Walk Test (F8W), and unconstrained walking (roaming) conditions (no exercise duration provided-likely about 10-minute)	1a) Creative novelty was rated by six experts on a scale of 1 (not original) to 5 (very original) for both experiments. Cronbach’s alpha was .79, and .70, respectively for the two experiments. 1b) Fluency and flexibility was scored by the primary investigator	1a) Novelty was highest in the roaming condition, compared to constrained walking and standing (*p* < .001). 1b) Fluency, flexibility, and novelty were highest in the roaming condition, compared to constrained walking and standing (*p* < .001). Constrained walking was also associated with higher fluency, flexibility, and novelty than standing (*p* < .001)

## Results

### Creativity Assessments

There are many different creativity assessments which may be utilized to experimentally assess acute creative potential in the laboratory. Four studies employed the Torrance Tests of Creative Thinking (TTCT) Figural Tests A and B (Torrance, Ball, & Safter, 1966), with one utilizing both the Figural and Verbal forms. Eight studies assessed divergent thinking, and two studies employed both divergent and convergent thinking assessments. Two studies evaluated analogy generation or production of metaphors. Assuming that these measures all demonstrate comparable quality in assessing certain aspects of creativity, caution should be observed when interpreting these results for practical generalizability.

### Study Description

Among the 13 manuscripts selected for this systematic review, all evaluated a hypothesized relationship between exercise manipulation and creativity performance. Of the 13 articles, three were published after 2013, two were published from 2002 to 2005, and eight were published from 1985 to 1998. Nine studies evaluated exercise and creativity within college-aged individuals, three studies assessed elementary and/or middle school children, and one study utilized a sample of adults at least 18 years of age. Nearly one-third of the included studies failed to report sample sizes per experimental or control group assignment. To this end, due to the substantial heterogeneity across study quality and methodology, a meta-analytic approach was not appropriate to include, and a qualitative review of research studies was chosen to avoid further convolution of conclusions suggested in the existing research on exercise and creativity (Walker, Hernandez, & Kattan, 2008). Three studies utilized a traditional within-subject design, four used a between-subjects protocol, one used a two-visit between-subjects design (Gondola, 1987), and four mixed-method studies implemented aerobic running sessions lasting 6–12 weeks, consisting of both between-and within-subject comparisons. One study was initially designed to employ a between-subjects design, but then collapsed the two experimental groups at the conclusion of the study to accrue a more robust sample size for analysis of treatment effects (Steinberg et al., 1997).

### Risk of Bias

Among the 13 experimental studies, 23% (*n* = 3/13) were determined to contain low risk of bias, 62% (*n* = 8/13) of the included studies were considered to be of moderate risk of bias, and 15% (*n* = 2/13) were considered to have been conducted with high risk of bias.

### Main Outcome Results Across Exercise Intensities

Among the 13 evaluated studies in this systematic review, 12 demonstrated some evidence of a beneficial effect of exercise on creativity. Further details on select studies, along with their limitations, are noted in the Discussion section. Among the 13 studies, eight evaluated moderate intensity and eight evaluated vigorous intensity exercise (two studies evaluated both moderate and vigorous intensity exercise). Regarding the eight studies focused on moderate intensity exercise, three demonstrated a significant effect of exercise on divergent thinking, specifically immediate and delayed improvements in figural creativity (Blanchette et al., 2005), increased fluency and novelty (defined as original and contextually appropriate) during and following exercise compared to rest, increases in high-quality analogies during exercise, an increase in novelty when walking outdoors (Oppezzo & Schwartz, 2014), as well as when roaming (free ambulation), or walking unconstrained. Notably, constrained walking was also shown to increase novelty compared to rest, but novel responses while walking along a predetermined path were statistically significantly lower than roaming unconstrained. A moderate intensity cycling protocol was shown to have no statistically significant influence on figural creativity measured by fluency, originality, and elaboration (although Curnow and Turner (1992) suggested weak support for a fluency effect at “the .05 level”). Among the seven studies employing vigorous exercise protocols, six demonstrated a significant effect. Convergent and divergent thinking was elevated among female dancers (although no scoring information was provided; Gondola, 1987). Alternatively, convergent thinking performance was reduced among inactive participants during intense cycling exercise, compared to both moderate intensity and rest conditions. Rest and moderate intensity cycling did not produce statistically significant outcomes in convergent thinking. Additionally, divergent thinking was higher during rest than intense exercise for inactive and active participants, with no statistically significant difference found between moderate cycling and rest (Colzato, Szapora, Pannekoek, & Hommel, 2013). Ramocki (2002) demonstrated statistical significance for divergent thinking fluency improvements following vigorous intensity exercise. This study was vulnerable to substantial bias, receiving a score of “1” on our quality assessment, so these findings should be cautiously interpreted. Steinberg et al. (1997) indicated divergent thinking flexibility was increased among participants; however, individuals completed either “low-impact, rhythmic stretching,” or “high-impact” aerobic dance, and were analyzed as a homogenous experimental group following the conclusion of the study. Therefore, those findings cannot be considered meaningful.

High intensity, chronic training studies (aerobic running durations of 20–30 minutes with 2–5 sessions per week) were shown to enhance divergent thinking performance (Gondola, 1986; Gondola & Tuckman, 1985; Herman-Tofler & Tuckman, 1998; Hinkle et al., 1993; Tuckman & Hinkle, 1986), with studies evaluating children demonstrating marginal gender-specific differences, specifically positing that girls may be more responsive to training-induced improvements in figural elaboration (Hinkle et al., 1993) and verbal flexibility and originality, with boys perhaps more likely to outperform girls on general divergent thinking measures (Tuckman & Hinkle, 1986).

## Discussion

### Results on Exercise and Creativity

Steinberg et al. (1997) evaluated aerobic dance and stretching lasting approximately 17–20 minutes in duration. The exercise protocol consisted of 6 minutes warm-up, 6 minutes of an aerobics class and 6 minutes of cool-down exercises. The aerobic exercise procedure was classified as “high impact,” while the rhythmic dancing was considered low-impact and consisted of a 4–5-minute warm-up, a 14-minute dance period, and 3–4 minutes of cooling down. Despite the decision to administer two distinct exercise protocols, the aerobics group and dancing group were combined into an aggregate sample following experimentation. This post-hoc deviation from the initial study design is problematic, as diverse modalities and intensities may uniquely influence the influence of physical exercise on creative cognitions. Creativity was also only measured post-exercise for this experiment, as further counterbalancing of creativity tasks would have rendered the procedure “too complicated and time-consuming.”

Other exercise protocols permitted participants to engage in weight lifting, as well as any accessible form of aerobic indoor or outdoor exercise (e.g., swimming, running, brisk walking, cycling, or stair climbing), continuous or intermittent, with a troubling dismissal of experimental control. Even among studies with adequate experimental control, the exercise regimen remained flawed, as one study asked participants to engage in both moderate and intense exercise within the same bout, failing to indicate that measurements of creative potential may be distorted by residual fatigue, particularly for inactive individuals randomized to complete the intense condition prior to the moderate intensity condition.

A crucial point must be considered as researchers aim to extend the field creativity and exercise. Specifically, the time-point at which creativity is assessed relative to the exercise bout warrants scrupulous empirical attention. If exercise is expected to exert evaluable effects on creative potential, then experiments must be designed to illuminate how, why, when, and for whom these effects may occur. Researchers often assess creativity before and after a single exercise bout (Curnow & Turner, 1992; Gondola, 1987; Ramocki, 2002) or multi-visit training program (Gondola, 1986; Gondola & Tuckman, 1985; Herman-Tofler & Tuckman, 1998; Hinkle et al., 1993). Although, some authors report testing creativity following the exercise bout, with no baseline assessment (Blanchette et al., 2005; Steinberg et al., 1997), while others administer a concurrent task protocol (Oppezzo & Schwartz, 2014; Zhou et al., 2017), to identify the relationship between creative cognitions activated during the transient stimulation of exercise. Research has also investigated timing differences between creativity assessed before and after, or during and immediately following acute exercise of moderate versus high-intensity (Colzato, Szapora, Pannekoek, & Hommel, 2013). There is utility in assessing potential temporal relationships across numerous research projects, or perhaps in a single research endeavor. To that end, it is troubling that no study, to date, has attempted to assess creativity before, during, immediately after, and many hours following exercise to test individual responses in a multi-visit study, in addition to isolating between-group differences contingent upon the timing of exercise.

### Informed Methods of Inquiry

All articles included reported that creativity was either augmented or decreased as a function of exercise manipulation, failing to underscore the reservations inherent in creativity tasks designed to evaluate specific creative correlates (i.e., divergent thinking, convergent thinking, insight, imagination, analogy, metaphor, etc.). Therefore, stating that creativity, *in general*, is improved or impaired by exercise, is as problematic as concluding that exercise improves or impairs a measure as intricate as intelligence, for science must always aim to specify precisely which outcome characteristics are changing in line with research interventions.

Three studies matched the duration of the creativity task with the duration of exercise. These studies employed between-subject designs, but the practice of time-matching creativity assessments to exercise stimulus in between-subject designs is a compelling direction for researchers to consider, especially when evaluating exercise-induced cognitive resource depletion, and/or residual effects of exercise persisting for a shorter creativity assessment, with a creativity measure requiring sustained mental resource allocation equivalent in length to the exercise bout.

Scoring of creativity tasks was inadequate for the majority of included manuscripts. Perhaps authors adhered to best research practices, such as use of a validated scoring manual, blinded rating, utilization of more than one rater, or using more objective statistical measures to denote the originality facet of creativity, such as identifying cut-point percentages, using the *top-three method*, or calculating a *creativity quotient* (Plucker, Qian, & Schmalensee, 2014; Plucker, Qian, & Wang, 2011; Silvia, Martin, & Nusbaum, 2009; Snyder, Mitchell, Bossomaier, & Pallier, 2004). It is crucial that participants be prohibited from scoring sample responses, even if these individuals are not scoring their own responses and are blinded to the identities of the other participants. One research study utilized this approach, following a creativity assessment protocol, administered in a group format, making rendering exclusive anonymity impossible. Other studies neglected to describe the scoring protocol, or the selection criteria and verification procedures used to identify *expert* scorers, which effectively obscures paths for subsequent research to follow in replication and refinement of methodological decisions.

### Limitations

Limitations of this review include the collaborative efforts of only two researchers to search databases and access relevant manuscripts. Searching three databases is another potential weakness, as it is possible research experiments fitting our inclusionary criteria may have been overlooked. However, we feel confident the search strategies employed were sufficiently comprehensive. Moreover, the full text of the exercise and creativity experiments extracted were read in full, and reference lists were crossed-checked by each of the primary researchers to ensure a parsimonious, yet extensive review of the literature was satisfied. Although quality assessment methods were developed in alignment with the PRISMA checklist for reporting systematic reviews, it is possible some items were overlooked which may have increased or reduced the bias scores for these studies. Further, the items developed to indicate risk of bias were formulated by the two researchers involved in this review. Additional researchers may have provided supplementary insight to refine the items to reflect higher quality evaluation methods than those achieved herein. Nevertheless, we feel the present evaluations are contextually appropriate, fair, and may engender continued discussion and more informed experimental practices.

### Strengths of Exercise and Creativity Research

Extant empirical investigations of the plausible relationship between exercise and creativity have provided a robust platform for continued exploration. Thus far, the field is beginning to depart from a general recognition of conjectural anecdotes suggesting physical movement may liberate mental constraints and encourage creative cognitions and is approaching an evidenced-based understanding. The articles included herein provide modest support for exercise to meaningfully impact creative thinking. Beyond the inclusion of various exercise modalities, durations, and environments (detailed in the methods section) by which to assess creativity, there are specific recommendations from well-conducted research that should inform future methods of inquiry in this arena.

Oppezzo and Schwartz (2014) theorized that an observed interplay between comfortable, self-selected ambulatory exercise and creativity may be associated with improvements in positive mood related to the mind-freeing nature of exercise and physical movement, which may also play a role in the activation of associative memory processes conducive to originality at the expense of conventional, ideation. Moreover, the authors additionally suggest that convergent thinking may require a higher degree of cognitive control, which would explain decrements in convergent thinking during acute exercise (Oppezzo & Schwartz, 2014). Relatedly, Colzato et al. (2013), examined the effects of exercise temporality and intensity on divergent and convergent thinking assessed both during and following acute exercise, demonstrating that sedentary individuals achieved higher convergent thinking during and after both moderate-intensity exercise and rest conditions compared to high-intensity exercise, and that habitual exercisers achieved higher flexibility in divergent thinking during rest compared to intense exercise, proposing that complex cognitions associated with convergent thinking and flexibly overcoming mental fixation may be vulnerable to depletion of cognitive control resources (Colzato, 2013).

### Recommendations for Future Research

Research has yet to definitively uncover *why* and *how* exercise may influence the global construct of cognitive creativity. However, the plausibility for exercise to exert measurable effects is encouraging, as facilitative mind-body connections have been extensively proposed as mechanisms for improvements in memory (Frith, Sng, & Loprinzi, 2017; Sng, Frith, & Loprinzi, 2017) and cognition (Chang, Labban, Gapin, & Etnier, 2012; Etnier et al., 1997; Loprinzi & Kane, 2015). In addition, much anecdotal evidence alludes to an influential relationship between physical exercise and cognitive creativity. Moreover, the underpinnings of movement and mental resource allocation are suggested to activate shared neural pathways, which further highlights the dynamic complexity of human physiology and cognition. Despite considerable efforts to illuminate this association, the results remain inconclusive. Future research should extend scientific understanding of such neural mechanisms in the context of exercise and creativity (Gonen-Yaacovi et al., 2013). Functional imaging of prefrontal areas of the human brain at rest, during a creativity assessment following a rest period, and during a creativity following exercise may facilitate a deeper understanding of neural activation subserving creative cognition. Additionally, baseline individual differences in complex-cognitions related to creativity (such as inhibition and flexibility), which may be captured using executive functioning assessments (Gilbert & Burgess, 2008), should be measured to determine whether baseline cognition mediates relationships observed between exercise and creativity thinking.

There appears to be weak to modest support for acute, moderate-intensity exercise to benefit creativity. High-intensity exercise appears to induce a detrimental effect on convergent creativity when the creativity task and vigorous exercise are administered concurrently in unfit individuals. Interestingly, rest, or the absence of exercise may have a similar deleterious influence on convergent creativity among regular exercisers, however subsequent research should attempt to further question these speculations by examining valence-related effects of exercising on creativity scores. Specifically, when habituated, and perhaps enjoyable behaviors (e.g., exercise) are withheld, is substitution of a less enjoyable activity (e.g., forced inactivity) in an environment conducive to exercise, capable of inducing negative affect or amotivation, which may act synergistically to reduce creative performance? Conversely, moderate-intensity exercise has also been shown to impair convergent thinking performance, suggesting that, perhaps, convergent tests of creativity are susceptible to exercise-driven depletion of mental resources necessary to complete the task, or reductions in attention, motivation, or affectual responses. Although, it is possible these speculations are entirely misguided, as one study found exercise may be capable of enhancing creativity, independently of changes in mood state. Nevertheless, results from the studies included herein tend to suggest a potential immediate and residual effect of exercise participation on creative performance, specifically divergent fluency and flexibility assessed in the laboratory, with improvements in divergent flexibility more equivocal. Oppezzo and Schwartz (2014) provided a practical interpretation of their findings, proposing that although walking may make people more talkative, fluency alone cannot equate to creativity. Therefore, the authors computed an additional analysis suggesting that appropriate novelty was elevated within individuals who walked, not only because these individuals were more fluent, but because their total ideation volume contained more divergent responses. To this end, it is prudent to consider the totality of the existing exercise and creativity research, fraught with shortcomings, but also promising trajectories for continued, careful investigation.

Aerobic training studies lasting at least 6 weeks in duration, and with at least two exercise session per week may have some utility on influencing adult and childhood creativity, however, these findings should be interpreted with caution, as many studies failed to employ a non-exercise control arm, or even standardize the exercise protocol within the experimental group. Additionally, one 8-week study showed no statistically significant improvements in 800-meter run performance, suggesting figural fluency was marginally augmented in the absence of fitness improvements. To date, it is unclear whether exercise benefits, undermines, or has no bearing on creative functioning. Therefore, research studies should focus on first identifying relationships in controlled laboratory environments, more robust to confounding factors unaccounted for in outdoor environments. Further, while it was unclear if creativity assessments were always administered in either an individualized or group setting. For all training studies, the exercise portion was completed in a group format, which may exert unintended effects on motivation, affect, and effort. All training studies included in this review failed to report one or all of the following statistical indices of practically meaningful results, including effect sizes, confidence intervals, or point estimates. Reliance on p-values is insufficient, incomplete, and misleading for any research agenda (Wilkinson, 2014). Moreover, none of the four training studies reviewed were conducted in a laboratory setting, which would be less of a limitation if compliance to the training protocol was detailed, or perhaps, if evidence for habitual exercise to benefit creative thinking was well established in the literature. Again, the vast majority of conclusions presented within exercise and creativity research deteriorate in plausibility as fragile study designs and analytic decisions are applied, perhaps for the purposes serving feasibility, but undeniably at the cost of scientific progress.

Despite the enigmatic challenges that emerge when assessing creativity in acute, laboratory settings, controlled measurement of creative potential is imperative for researchers to accumulate a comprehensive understanding of the various manipulations designed to address proposed associations between exercise and creativity. Controlled, empirical work will allow researchers to provide compelling evidence for theoretical mechanisms underlying the proposed exercise-creativity link. We suggest that an exercise-driven approach to measuring creativity is an exciting avenue for continued scientific investigation of the longitudinal effects of exercise on the creative person, including motivation and personality factors (Cropley, 2000), as well as acute and chronic effects of exercise on creativity performance across various age groups.

Future research is warranted to assess the influence of physical activity in early childhood on movement-based creativity outcomes, such as Torrance’s Thinking Creatively in Action and Movement Test (TCAM), which was designed on the precedent that young children manipulate and organize their thoughts in expressive, kinesthetic actions, as their proficiency in verbalization, writing, or drawing may be less cultivated at the preschool age (Torrance, 1981). Regarding the experiments reviewed herein, three provided evidence for chronic physical exercise to confer higher figural and verbal creativity (measured via the TTCT and AUT) in children between the third and sixth grade (Herman-Tofler & Tuckman, 1998; Hinkle & Sampson, 1993; Tuckman & Hinkle, 1986). To our knowledge, no acute exercise studies have been conducted in childhood populations. Further, exercise and creativity research extended to younger populations is warranted. Notably, however, young children may not provide responses that accurately reflect their creative capacity in words or drawings, but, rather, are perhaps more likely to act out their thoughts using symbolic, representational movement (Zachopoulou, Makri, & Pollatou, 2009). Torrance proposed that bodily motion is an indelibly potent strategy for unveiling creativity in early childhood populations (Torrance, 1981). Thus, it is worthwhile for researchers to consider that physical activity may promote creative, associative thinking, particularly in physical domains, perhaps representing an interface between tactile, sensorimotor representations of developing complex cognitions.

Research investigating the utility for physical activity to influence creative cognitions in older adulthood is also warranted. Fifty-nine years is the oldest age reported to have been assessed in the exercise and creativity experiments reviewed herein, with the age range of that experiment ranging from 19 to 59 (Steinberg et al., 1997). Future work focusing specifically on potential exercise-induced influences on creativity in aging populations is important. Previous search suggests that aging individuals with dementia generally exhibit impaired performance on laboratory assessments of divergent thinking (Cruz de Souza et al., 2010; Hart & Wade, 2006). Perhaps regular exercise may attenuate dementia symptomology and aging-induced frontal-lobe dependent decrements in cognition (Colcombe & Kramer, 2003), as the prefrontal cortex shares functional neural connections with motor regions in the brain (Cole et al., 2013), which may facilitate the maintenance of higher-order mental functions, including creativity, in later life.

## Conclusions

Weak evidence exists, to date, in support of the proposed relationships between exercise and creative thinking processes. Inferences of causality are difficult to accept, given the paucity of well-designed experiments in this domain of scientific investigation. Exercise and creativity researchers should first align their methodologies with unbiased measurement and evaluation practices, carefully designed to answer prudent explanatory questions. Restructuring the current framework requires a swift dismissal of ideological barriers to discovery, namely the conflation of creativity with divergent thinking, as well as unmitigated advancement into the dense tangle of speculative discourse aiming to contrive tenuous links between creativity and exercise. Experiments continue to employ minimal standardization, laboratory control, resistance to confounding, and rigorous, detailed scoring procedures, leaving the same questions unanswered and limiting valid conclusions. The prospects for growth and development in research examining creativity and exercise associations are astronomical, but only if the field commits to consistency and quality when assessing the potential for such relationships.
